# Effect of modified Laprade technique on posterolateral ligament injury of knee

**DOI:** 10.1186/s40001-022-00764-2

**Published:** 2022-09-07

**Authors:** Kaibin Fang, Zhangsheng Dai, Xiaocong Lin

**Affiliations:** Department of Orthopaedic Surgery, The Second Affiliated Hospital of Fujian Medical University, No.34, Zhongshanbeilu, Quanzhou, 36200 Fujian China

**Keywords:** Modified Laprade technique, Posterolateral structure injury, Anatomical reconstruction

## Abstract

**Purpose:**

To investigate the effect of modified Laprade technique on the reconstruction of posterolateral structure of knee and anterolateral ligament of knee in the treatment of posterolateral injury of knee.

**Methods:**

From December 2013 to June 2020, multiple ligament injury patients who received surgery in our hospital were collected in this research. These patients underwent a modified Laprade technique for posterolateral structural reconstruction of the knee. Lysholm scores of patients pre- and post-operation were recorded.

**Result:**

The operations of the observation group or the control group patients were completed. There were no significant differences in gender, age, preoperative knee range of motion and preoperative Lysholm score. At the time of follow-up 1 month after operation, there was no significant difference in knee range of motion, dial-up test angle and Lysholm score between the observation and the control group. When followed up 1 year after operation, the Lysholm score of the observation group was higher than that of the control group. The difference was statistically significant. The same situation occurred in the range of motion of the knee in both groups. However, there was still no significant difference between the two groups in the dial-up test 1 year after operation, whether the knee flexion was 30° or 90°.

**Conclusion:**

For patients with posterolateral structure injury of knee, the modified Laprade technique is a feasible surgical technique.

## Introduction

Posterolateral structure of knee was a stable structure with complex anatomy and function, composed of multiple tendons and ligaments [1]. The injury of this structure will cause serious knee dysfunction, such as knee varus, tibial external rotation and tibial recoil [2].The injury of this structure was mostly accompanied with the injury of cruciate ligament and anterolateral ligament injury. In the reconstruction of cruciate ligament injury, if the treatment of posterolateral structure injury of knee was ignored, reconstruction would failed [3]. Laprade method was a very classic surgical method for the treatment of posterolateral structural injury of knee [4]. Through this operation, the important posterolateral structures of the knee could be anatomically reconstructed. The author tried to repair the anterolateral ligament based on Laprade method, and changed the implantation direction of fibular screw.

## Materials and methods

**Fig. 1:**
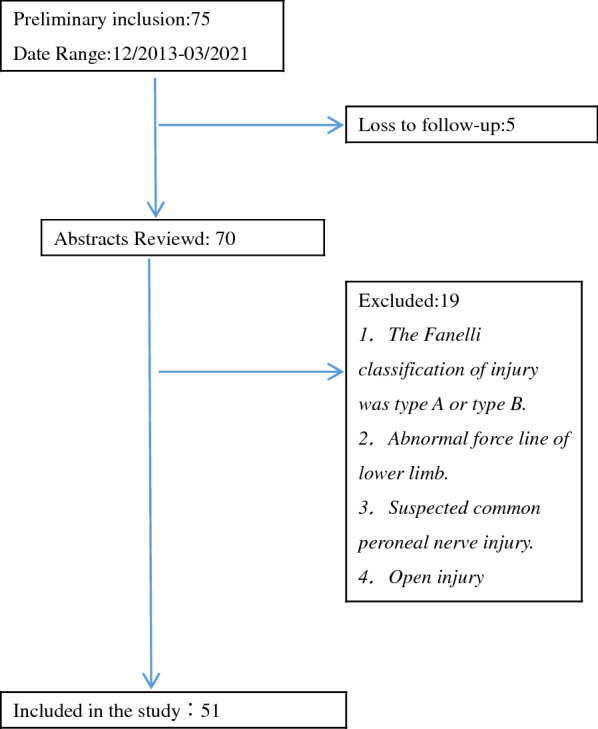
The exclusion criteria and inclusion criteria

This was a retrospective study from December 2013 to June 2020. Patients with posterolateral structural injury who met the inclusion criteria (age more than 18 years) were included in the study. The exclusion criteria and inclusion criteria are shown in Fig. 1. All patients underwent magnetic resonance imaging. Fanelli typing was used to evaluate the injury of patients [5]. Patients with Fanelli type C needed surgery. A total of 51 patients were included in the study, 10 patients underwent the traditional Laprade method for surgery, considered to be the control group. 11 patients underwent the modified Laprade method for surgery, considered to be the observation group. The operations of all patients were performed by the same orthopaedic team. The chief surgeon has 20 years of surgical experience.

**Fig. 2:**
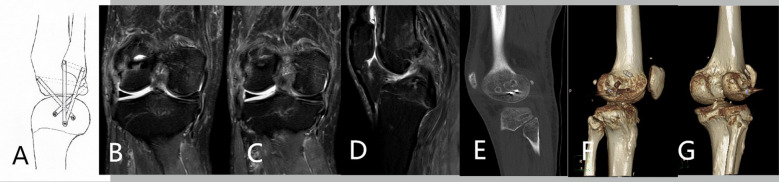
This was a 49-year-old male patient who underwent posterior cruciate ligament reconstruction in another hospital and still had knee relaxation. The patient then underwent a modified Laprade technique to reconstruct the posterolateral structure. **A** Operation diagram. **B**, **C**, **D** Preoperative imaging data. **E**, **F**, **G** Imaging data after operation

**Fig. 3:**
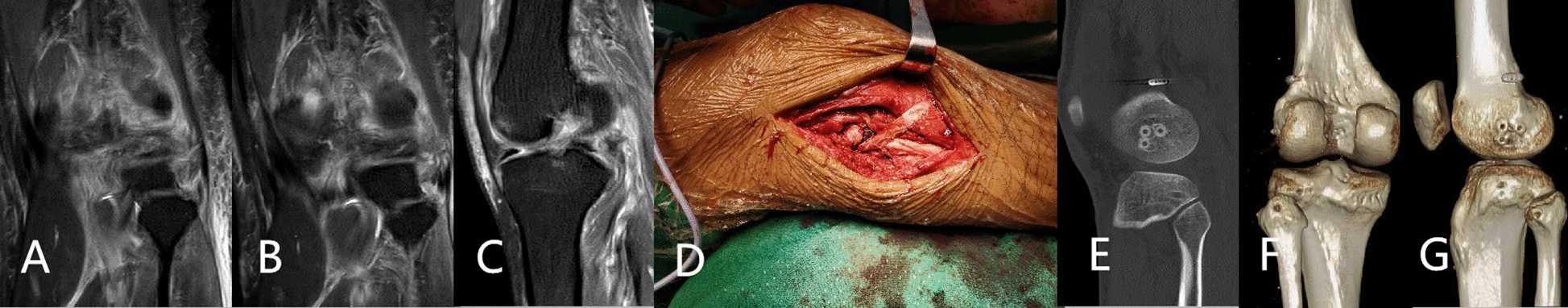
This was a 39-year-old female patient with posterior cruciate ligament and posterolateral structural injury. The patient then underwent a modified Laprade technique to reconstruct the posterolateral structure. **A**, **B**, **C** Preoperative imaging data. **D** Intraoperative pictures. **E**, **F**, **G** Postoperative imaging data

The classic Laprade method was used in the operation of patients in the control group. The posterolateral structures of these patients were reconstructed by the transfer of their own tendons. The patients in the observation group underwent surgery using the modified Laprade method. First the patient underwent arthroscopic anterior cruciate ligament reconstruction. During the operation of the patients in the observation group, a half tendon of the peroneus longus was removed as a graft. The lateral side of the knee was cut about 12 cm, so that the patient's lateral collateral ligament was fully exposed. The distal end of the incision was at the midpoint of the line between Gerdy’s nodule and fibular head. The proximal end of the incision was parallel to the posterior edge of the iliotibial tract. The common peroneal nerve can also be exposed and protected through this incision. A 6-mm bone canal was drilled at the attachment point of the lateral collateral ligament towards the posterior side of the lateral epicondyle of the femur. Then, a 6-mm bone canal was drilled at the attachment point of the lateral condyle of the hamstring tendon to the front of the lateral epicondyle of the femur. The common peroneal nerve can then be pulled back for protection. This was because the operator needs to drill a 6-mm bone tunnel from front to bottom to back at the maximum diameter of the fibular head. After that, a 6-mm bone tunnel was drilled from about one transverse finger within the Gerdy tubercle of the tibia from front to back to the posterolateral tibial plateau. As a graft, the autologous tendon was divided into two parts, which were implanted into the femoral bone canal and fixed by compression nail. One of the tendons to be used as the lateral collateral ligament was inserted into the fibular tunnel. The compression nail at the fibular tunnel should be screwed in from the back up to the front down. The tendon passing through the fibular tunnel was led forward from the tunnel behind the tibia together with the other tendon, and then fixed in front of the tibial plateau. The other tendon was used to reconstruct the popliteal tendon. In this way, the popliteal peroneal ligament and popliteal tendon were reconstructed. The tendon used to reconstruct the popliteal tendon has some residual after reconstruction. This residual tendon can be sutured and fixed at the upper back of the lateral collateral ligament from the Gerdy tubercle to the outside. This was to reconstruct the anterolateral ligament. The procedure and typical cases are shown in Fig. 2 and Fig. 3.

The knees of all patients were fixed at 15° flexion by brace after operation. The pillow was placed under the patient’s knee to avoid backward movement of the tibia. Patients were encouraged to perform isometric contraction of quadriceps femoris and straight leg lifting after operation. 6 weeks after surgery, the patient was allowed to flex the knee. The patient was allowed to partially load under the protection of brace 1 months after operation. At 3 months after surgery, the patient was allowed to remove the brace and carry the weight completely. Half a year after surgery, the patient was allowed to gradually resume physical activity.

### Statistical analysis

Statistical assessment was performed using SPSS V19 software. Consecutive data were summarized as mean and standard deviation (SD), or median and range; whereas categorical data were summarized as frequencies and percentage. Comparison among categorical variables was performed using Chi-square test; for continuous data, independent *t*-test was used if variables were normally distributed, otherwise Mann–Whitney test was employed.

## Results

The operations of the observation group and the control group patients were successfully completed. The incisions of these patients healed, and there were no complications such as wound infection, neurovascular injury and lower extremity deep venous thrombosis. All patients were followed up for more than 1 year. There were no significant differences in gender, age, preoperative knee range of motion and preoperative Lysholm score. At the time of follow-up 1 month after operation, there was no significant difference in knee range of motion, dial-up test angle and Lysholm score between the observer and the control group. When the patients were followed up 1 year after operation, the Lysholm score of the observation group was higher than that of the control group. The difference was statistically significant. The same situation occurred in the range of motion of the knee in both groups. However, there was still no significant difference between the two groups in the dial-up test 1 year after operation, whether the knee flexion was 30° or 90°. The postoperative functional comparison between the two groups is shown in Table 1.

**Table 1:** Demographic data and clinical outcomes

Variable	Modified group (*n* = 21)	Control group (*n* = 30)	Statistic(*T*-value/*χ*^2^-value)	*p*-value
Gender(male/female)	(15/6)	(19/11)	0.36	0.55
Age (year)	39.27 ± 6.33	37.65 ± 9.03	0.71	0.48
Preoperative knee range of motion(°)	71.55 ± 9.56	73.31 ± 10.35	0.62	0.54
Knee range of motion(°) 1 month after operation	103.27 ± 9.11	101.26 ± 10.05	0.73	0.47
Comparison of knee joint activity before operation and 1 month after operation				
Statistic (*T*-value/*χ*^2^-value)	12.08	10.79		
* p*-value	< 0.01	< 0.01		
Knee range of motion(°) 1 year after operation	117.56 ± 7.33	106.25 ± 9.19	4.69	< 0.01
Preoperative Lysholm score	45.96 ± 7.03	47.29 ± 9.33	0.55	0.58
Lysholm score 1 month after operation	80.73 ± 5.33	81.09 ± 6.05	0.22	0.83
Comparison of Lysholm score before operation and 1 month after operation				
Statistic (*T*-value/*χ*^2^-value)	0.52	16.65		
* p*-value	0.60	< 0.01		
Lysholm score 1 year after operation	83.75 ± 6.27	79.52 ± 5.16	2.64	0.01
Preoperative Dial test				
Knee flexion 30°	13.75 ± 1.03	13.96 ± 1.11	0.68	0.50
Knee flexion 90°	14.77 ± 1.63	15.05 ± 1.67	0.60	0.55
Dial test 1 month after operation				
Knee flexion 30°	5.63 ± 1.05	5.71 ± 1.23	0.24	0.81
Knee flexion 90°	6.35 ± 1.33	6.27 ± 1.15	0.14	0.89
Dial test 1 year after operation				
Knee flexion 30°	5.33 ± 1.16	5.23 ± 1.65	0.24	0.81
Knee flexion 90°	6.27 ± 1.58	6.15 ± 1.36	0.28	0.77

Values are presented as mean ± SD

Table 1 Comparison of postoperative function between the two groups.

## Discussion

Knee is the largest and most complex joint in human body. Its good motor function and stable state depend not only on the bone structure, but also on the ligament stability structure. The injury of posterolateral structure often caused serious instability of knee, resulting in limited function of knee. In the long term, it can cause irreversible damage to cartilage and other bone structures [6].The posterolateral structure injury did not occur simply, it is often accompanied with the posterior cruciate ligament injury [7]. However, posterolateral angle injury is often ignored. Simply reconstructing the posterior cruciate ligament and ignoring the reconstruction of the posterolateral angle will cause varus and external rotation of the knee, resulting in instability of the knee [8]. Laprade method was a very effective anatomical reconstruction method [9]. The ligament reconstructed by this method has good biomechanical properties [10]. However, this method only anatomically reconstructed the important posterolateral structures, and did not reconstruct the anterior structures such as anterior cruciate ligament and anterolateral ligament. This may easily lead to instability of the front side or instability of the anterolateral rotation, and residual varus relaxation to a certain extent.

In this study, the direction of fibular screw placement was improved. At the same time, the anterior structure was strengthened while reconstructing the posterolateral structure. The patients who underwent modified reconstruction had better knee function and functional score as patients who underwent traditional reconstruction in the early postoperative period. However, at the time of follow-up 1 year after operation, patients treated with the modified method showed advantages in knee range of motion and function score. It suggested that the modified method was more effective. The placement direction of fibular screw was changed from the posterior direction of fibula to the anterior direction of fibula, which can tighten the tendon. This is because both screws and tendons compete for space in the bone tunnel. Placing fibular screws from front to back allows the tendon to extend outward. The tendon is therefore tensioned.

Cruciate ligament and collateral ligament are important structures to maintain the stability of knee. However, the importance of extra articular structures should not be ignored. Like the posterolateral structure, the anterolateral structure also makes an important contribution to the stability of anterior direction [11]. In our modified method, the residual transplanted tendon was placed anterolaterally. In this way, the anti-rotation ability of the knee is enhanced. Injury to the anterolateral structure of the knee can also lead to knee instability, which is often not found at the first time [12]. The anterolateral structure mainly provides help for the full function and stability of the anterior cruciate ligament [13].In previous work, the author found that some patients may still have knee instability after traditional Laprade reconstruction, mainly anterolateral instability of the knee. After the improvement of the surgical method, the stability of the patient's knee was improved. On the other hand, it also suggests that patients with posterolateral Structure Injury of knee may often be combined with anterolateral Structure Injury of knee. But this conjecture still needs more case studies to confirm.

Based on the current research, the author believed that for patients with posterolateral structure injury of knee, the modified Laprade technique was a feasible surgical technique.

## Data Availability

All data included in this study are available upon request by contact with the corresponding author.

## References

[1] Ullrich K, Krudwig WK, Witzel U (2002). Posterolateral aspect and stability of the knee joint. I. Anatomy and function of the popliteus muscle-tendon unit: an anatomical and biomechanical study. Knee Surg Sports Traumatol Arthrosc.

[2] Krudwig WK, Witzel U, Ullrich K (2002). Posterolateral aspect and stability of the knee joint .II. Posterolateral instability and effect of isolated and combined posterolateral reconstruction on knee stability: a biomechanical study. Knee Surg Sports Traumatol Arthrosc.

[3] Kinsella SD, Rider SM, Fury MS (2019). Concomitant posterolateral corner injuries in skeletally immature patients with acute anterior cruciate ligament injuries. J Pediatr Orthop.

[4] Laprade RF, Resig S, Wentorf F (1999). The effects of grade III posterolateral knee complex injuries on anterior cruciate ligament graft force. A biomechanical analysis. Am J Sports Med.

[5] Fanelli GC, Feldmann DD (1999). Management of combined anteriorcruciate ligament/posterior cruciate ligament/posterolateral complex injuries of the knee. Op Tech Sports Med.

[6] Laprade RF (2002). The effect of injury to the posterolateral structures of the knee on force in a posterior cruciate ligament graft. Am J Sports Med.

[7] Malone AA, Dowd G, Saifuddin A (2006). Injuries of the posterior cruciate ligament and posterolateral corner of the knee. Injury.

[8] Lutz PM, Merkle M, Winkler PW (2021). Combined posterolateral knee reconstruction: ACL-based injuries perform better compared to PCL-based injuries. Knee Surg Sports Traumatol Arthrosc.

[9] Laprade RF, Johansen S, Wentorf FA (2004). An analysis of an anatomical posterolateral knee reconstruction: an in vitro biomechanical study and development of a surgical technique. Am J Sports Med.

[10] Laprade RF, Spiridonov SI, Coobs BR (2010). Fibular collateral ligament anatomical reconstructions: a prospective outcomes study. Am J Sports Med.

[11] Ferretti A, Monaco E, Fabbri M (2017). Prevalence and classification of injuries of anterolateral complex in acute anterior cruciate ligament tears. Arthroscopy.

[12] Musahl V, Rahnemai-Azar AA, Costello J (2016). The influence of meniscal and anterolateral capsular injury on knee laxity in patients with anterior cruciate ligament injuries. Am J Sports Med.

[13] Noyes FR, Huser LE, Jurgensmeier D (2017). Is an anterolateral ligament reconstruction required in ACL-reconstructed knees with associated injury to the anterolateral structures?. Am J Sports Med.

